# Impact of *Bacillus cereus* on the Human Gut Microbiota in a 3D In Vitro Model

**DOI:** 10.3390/microorganisms11071826

**Published:** 2023-07-17

**Authors:** Marco Calvigioni, Adelaide Panattoni, Francesco Biagini, Leonardo Donati, Diletta Mazzantini, Mariacristina Massimino, Costanza Daddi, Francesco Celandroni, Giovanni Vozzi, Emilia Ghelardi

**Affiliations:** 1 Department of Translational Research and New Technologies in Medicine and Surgery, University of Pisa, 56127 Pisa, Italy; marco.calvigioni@med.unipi.it (M.C.);; 2 Department of Information Bioengineering, University of Pisa, 56126 Pisa, Italygiovanni.vozzi@unipi.it (G.V.); 3 Research Centre “Enrico Piaggio”, University of Pisa, 56126 Pisa, Italy

**Keywords:** in vitro model, gut microbiota, gut microbes, intestinal communities, *Bacillus cereus*, gastrointestinal infection

## Abstract

In vitro models for culturing complex microbial communities are progressively being used to study the effects of different factors on the modeling of in vitro-cultured microorganisms. In previous work, we validated a 3D in vitro model of the human gut microbiota based on electrospun gelatin scaffolds covered with mucins. The aim of this study was to evaluate the effect of *Bacillus cereus*, a pathogen responsible for food poisoning diseases in humans, on the gut microbiota grown in the model. Real-time quantitative PCR and 16S ribosomal RNA-gene sequencing were performed to obtain information on microbiota composition after introducing *B. cereus* ATCC 14579 vegetative cells or culture supernatants. The adhesion of *B. cereus* to intestinal mucins was also tested. The presence of *B. cereus* induced important modifications in the intestinal communities. Notably, levels of *Proteobacteria* (particularly *Escherichia coli*), *Lactobacillus*, and *Akkermansia* were reduced, while abundances of *Bifidobacterium* and *Mitsuokella* increased. In addition, *B. cereus* was able to adhere to mucins. The results obtained from our in vitro model stress the hypothesis that *B. cereus* is able to colonize the intestinal mucosa by stably adhering to mucins and impacting intestinal microbial communities as an additional pathogenetic mechanism during gastrointestinal infection.

## 1. Introduction

In the last few decades, studying the human gut microbiota has become one of the hottest topics in scientific research. Clinical studies and different animal models have traditionally been used to evaluate the composition and functionalities of intestinal microbial populations in healthy and pathological conditions [1,2]. In parallel, increasingly modern, technological, and diversified in vitro models to culture complex microbial communities, such as the gut microbiota, have been developed and gradually validated as possible alternatives for studying microbial consortia residing in the gut ex vivo [3,4]. Reproducibility, easy access, and the possibility of continuous monitoring are just some of the advantages displayed by in vitro models compared to animal models or human individuals. Among the several application possibilities, in vitro models supplemented with pathogenic microorganisms can easily be used to evaluate the effects of certain pathogens on the in vitro-cultured microbiota [5,6,7]. Therefore, this approach aims to resemble in vivo infections as much as possible. Nevertheless, this approach is still uncommon, and only a few studies involving in vitro-grown gut microbiota in co-culture with intestinal pathogens are present in the literature [5,6,7]. In particular, the impact of enterotoxigenic *Escherichia coli* [5,6] and *Clostridioides difficile* [7] on the composition of gut communities has been investigated to date.

In this study, a previously validated three-dimensional (3D) in vitro model of the human gut microbiota [8,9,10] was used. The model is based on electrospun reticular gelatin scaffolds suitable for enhancing microbial adhesion and biofilm formation and preserving the richness and biodiversity of the in vitro-cultured gut microbes, as reported by Biagini et al. [8,9]. It was also shown that adding a mucin coat to the electrospun structures selects intestinal microorganisms able to adhere to mucins following adaptative behaviors in the presence of mucus [10].

The developed 3D in vitro system was used to evaluate the effect of an enterotoxigenic *Bacillus cereus* strain on the cultured gut microorganisms. *B. cereus* is a Gram-positive, rod-shaped, spore-forming bacterium primarily responsible for food poisoning and severe extra-intestinal infections in humans and mammals [11,12]. Due to its ability to resist harsh environments by forming spores and biofilms, *B. cereus* is globally distributed, especially in soil and vegetation. Although outbreaks have been reported worldwide in the last few decades [13,14,15,16,17], the prevalence of food poisoning and gastrointestinal infections caused by *B. cereus* is underestimated and uncertain [18]. Besides producing cereulide in contaminated foods, which is responsible for the emetic syndrome [19], *B. cereus* can colonize the human intestinal tract and produce other virulence factors determining the diarrheal syndrome [11,18,19]. However, the impact of *B. cereus* on the microbial communities inhabiting the gut is unknown. In this work, we aimed to evaluate the ability of *B. cereus* ATCC 14579 to adhere to mucins in vitro and the effects of *B. cereus* vegetative cells and culture supernatants on the in vitro-grown intestinal communities.

## 2. Materials and Methods

### 2.1. Fecal Microbiota and Bacterial Strains

A voluntary human donor of fecal samples was selected, as previously detailed [8]. Written informed consent was obtained by the donor before starting the study. Stools were processed following the European Guidelines for Fecal Microbiota Transplantation [20]. Aliquots of fecal suspension were stocked at −80 °C in 10% *v/v* glycerol until use. *Bacillus cereus* ATCC 14579 was used as a reference strain in this study due to its well-characterized pathogenicity and virulence profile [21] and ability to form biofilm [22].

### 2.2. In Vitro Adhesion of B. cereus to Mucins

Adhesion to mucins was assessed as previously described by Mazzantini et al. [23], with slight modifications. *B. cereus* was streaked on Brain Heart Infusion (BHI, Thermo Fisher Scientific, Waltham, MA, USA) agar plates and incubated at 37 °C for 24 h. Isolated colonies were separately inoculated in 5 mL of Roswell Park Memorial Institute (RPMI) 1640 medium (without L-glutamine and phenol red, Merck KGaA, Germany). A total of 100 μL of overnight cultures were inoculated in 25 mL of fresh RPMI 1640 and grown to an optical density at 600 nm (OD_600_) of 1.5. Suspensions were centrifuged for 10 min at 3900 rcf at 4 °C, and pellets were washed two times with sterile phosphate-buffered saline (PBS, 1 M KH_2_PO_4_, 1 M K_2_HPO_4_, 5 M NaCl, pH 7.2). PBS was added to reach an OD_600_ of 1.5 (≈ 10^8^ cells/mL). A total of 500 μL of the suspensions were added to 48-well microplates with a flat bottom (Thermo Fisher Scientific) containing 600 μL of mucin agar, constituted by 5% *w/v* of mucins from porcine stomach type II (Merck KGaA) and 1% *w/v* of bacteriological agar. As negative controls, 500 μL of the same microbial suspensions were inoculated on wells containing 600 μL of 1% *w/v* bacteriological agar without mucins. Plates were incubated at 37 °C for 30 min in static conditions, followed by 60 min gentle, constant shaking (50 rpm). After incubation, supernatants were discarded, and wells were washed two times with 500 μL of PBS to remove superficially loosely adhered cells. Microbial extraction from agar was performed by mechanical methods. The agar cylinders were aseptically transferred into 5 mL of physiological peptone solution and mechanically homogenized for 5 min. Aliquots were seeded on BHI agar plates to quantify adhered bacteria by the plate count method.

### 2.3. Preparation of Mucin-Coated Electrospun Gelatin Structures

Electrospun gelatin (EG) structures were biofabricated, cut, and sterilized, as previously reported [9]. Sterile scaffolds were separately inserted in 24-well microplates with a flat bottom (Thermo Fisher Scientific). A total of 200 µL of a previously autoclaved solution made of 5% *w/v* of mucins (type II mucins from the porcine stomach, containing MUC2, Merck KGaA) in water were added to each well of the EG structures and microplates incubated overnight at 4 °C [9]. After incubation, wells were washed three times with 1 mL of sterile PBS, and mucin-coated electrospun gelatin (EGM) structures were used as scaffolds to sustain microbial growth throughout the study.

### 2.4. Preparation of B. cereus Suspensions and Culture Supernatants

An isolated colony of *B. cereus* from BHI agar plates was inoculated in 5 mL of RPMI 1640 and incubated overnight at 37 °C. A total of 100 µL of the overnight culture were inoculated in 5 mL of fresh RPMI 1640 and grown up to an OD_600_ of 1.2 (≈5 × 10^5^ cells/mL). The suspension was centrifuged for 10 min at 3900 rcf at 4 °C. The supernatant was collected, filtered using syringe-driven 0.22 µm filters (JetBiofil, China), and transferred to a sterile tube. Pellets were washed two times with PBS and suspended in RPMI 1640 up to an OD_600_ of 1.2. *B. cereus* supernatant and fresh suspension with vegetative cells were immediately used to inoculate plates prepared as described in Section 2.5.

### 2.5. In Vitro Microbiota Culture on the EGM Scaffolds and Addition of B. cereus

A total of 100 µL of fecal suspension, prepared as reported in Section 2.1, was inoculated upon sterile EGM structures in each well of the 24-well microplates. A total of 1.9 mL of RPMI 1640 was added to a final volume of 2 mL. Plates were incubated for 24 h at 37 °C in an anaerobic atmosphere and generated using AnaeroGen^TM^ Compact (Thermo Fisher Scientific). After incubation, 100 µL of supernatants were removed from all wells. Half of the wells were inoculated with 100 µL of the suspension of *B. cereus* prepared as described above (Section 2.3). In the remaining wells, 100 µL of *B. cereus* supernatant obtained in Section 2.3 were added. Plates were incubated for 24 h at 37 °C in an anaerobic atmosphere. After incubation, culture supernatants (2 mL) were removed from all wells and replaced with 2 mL of fresh RPMI 1640. This grossly eliminates planktonic *B. cereus* vegetative cells and their secreted virulence factors. Plates were incubated for a further 24 h at 37 °C in an anaerobic atmosphere. In parallel, sterility control wells were included, constituted by sterile EGM structures and RPMI 1640 medium without fecal samples and *B. cereus* vegetative cells and supernatant. After incubation, supernatants were discarded, and EGM scaffolds were collected for DNA extraction.

### 2.6. Genomic DNA Extraction

As previously reported, microbial DNAs were extracted from EGM scaffolds using the phenol–chloroform method [10]. Extracted DNAs were quantified using NanoDrop Lite Spectrophotometer (Thermo Fisher Scientific) and normalized to a standard 5 ng DNA/µL concentration.

### 2.7. Real-Time Quantitative PCR

Absolute abundances of total bacterial load and the main phyla (i.e., Firmicutes, Bacteroidetes, Actinobacteria, and Proteobacteria) and genera (i.e., Akkermansia, Bacillus, Bacteroides, Bifidobacterium, Clostridium, Escherichia-Shigella, Faecalibacterium, Lactobacillus, Prevotella, and Ruminococcus) were assessed on extracted DNAs by 16S ribosomal RNA (rRNA) gene-targeting Real-time quantitative PCRs (qPCRs). Different primer pairs targeting phylum- or genus-specific regions of the gene encoding for the 16S rRNA are listed in Tables S1 and S2 (Supplementary Materials). A primer pair targeting a sequence of the 16S rRNA gene conserved in all bacteria was also used to evaluate the total bacterial abundance (Table S1). qPCR reactions were performed using the CFX96 Real-Time System (BioRad, Hercules, CA, USA) and CFX Maestro Software (version 2.3, BioRad). All reactions were carried out in duplicate in a 96-well plate with a final volume of 20 µL per well, including 8 µL of sterile water, 0.5 µL of each primer (10 µM), 10 µL of Luna Universal qPCR Master Mix (New England BioLabs, Ipswich, MA, USA), and 1 µL of 5 ng DNA/µL template DNA. The amplification protocol was as follows: an initial denaturation step at 95 °C for 1 min, followed by 45 cycles comprising a denaturation step at 95 °C for 15 s, an annealing step at each primer set specific temperature (Tables S1 and S2) for 30 s, and an extension step at 72 °C for 10 s. Absolute quantifications were performed by comparing calibration curves generated using external standards with known concentrations subjected to serial 10-fold dilutions ranging from 10^2^ to 10^10^. For each standard curve, the R^2^ coefficient was higher than 0.98.

### 2.8. 16S rRNA Gene Sequencing and Metagenomic Analysis

16S rRNA gene sequencing and subsequent data processing were performed by Novogene (Beijing, China). 16S rRNA gene regions V3–V4 were amplified with primers 341F (5′-CCTAYGGGRBGCASCAG-3′) and 806R (5′-GGACTACNNGGGTATCTAAT-3′). PCR products were detected with a 2% agarose gel electrophoresis and purified with the QIAGEN Gel Extraction Kit (QIAGEN, Germany). Sequencing libraries were generated using the NEBNext Ultra DNA Library Prep Kit for Illumina (New England BioLabs). Their quality was evaluated with the Qubit@2.0 Fluorometer (Thermo Fisher Scientific) and the BioAnalyzer 2100 System (Agilent Technologies, Santa Clara, CA, USA). Libraries were sequenced on the HiSeq Illumina platform, and 250 bp reads were generated. Raw data were filtered using QIIME2. Operational Taxonomic Units (OTUs) were clustered with a ≥97% similarity cut-off using Uparse. Representative sequences of each OTU were then analyzed using the GreenGene database based on the RDP classifier algorithm. Phylogenetic relations between OTUs were assessed with MUSCLE, and α-diversity analysis was performed using QIIME2 and R.

### 2.9. Statistical Analysis

Three biological replicates, with two technical replicates each, were performed. All statistical analyses were performed using GraphPad Prism (version 9.5.0, Dotmatics, Boston, MA, USA). Statistical significance was set at a *p*-value < 0.05. Data are expressed as the mean ± standard deviation. A Student’s t-test for unpaired data was applied for the adhesion assay. For Real-time qPCR experiments and α-diversity indexes, a one-way ANOVA followed by a Tukey post-hoc test for multiple comparisons was performed to compare results obtained from EGM scaffolds alone and EGM scaffolds in the presence of *B. cereus* or its culture supernatant at each time of incubation.

## 3. Results

### 3.1. B. cereus Adhesion to Mucins

**Figure 1:**
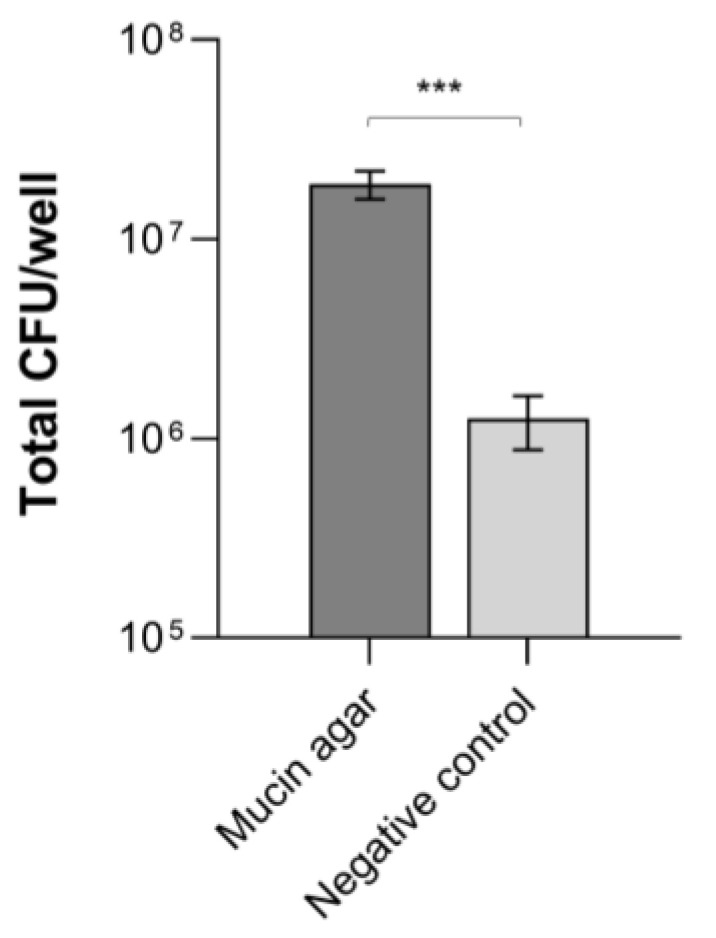
Adhesion (CFU/well) of *B. cereus* ATCC 14579 to mucins. *** *p* < 0.001.

Since the electrospun structures used as scaffolds for microbial growth in the in vitro model were covered with a mucin coat, the ability of *B. cereus* ATCC 14579 to adhere to mucins was first evaluated. As shown in Figure 1, the number of *B. cereus* vegetative cells recovered from mucin agar was significantly higher than that obtained from wells containing agar alone (i.e., a negative control) (*p* = 0.0005). This result indicates that *B. cereus* is able to bind and strongly adhere to mucins in vitro. This suggests that adhesion to mucins may also be important in vivo for *B. cereus* to temporarily colonize the gastrointestinal mucosa during infection.

### 3.2. Absolute Quantification of Bacterial Populations by Real-Time qPCR

To test the effect of *B. cereus* or its secreted compounds on the intestinal microbial communities, *B. cereus* vegetative cells (BC) or culture supernatants (S) were inoculated on the in vitro-grown gut microbiota. Genomic DNAs extracted from microbial consortia grown on scaffolds were thus subjected to 16S rRNA gene targeting qPCRs to obtain quantitative information on the composition of the gut microbiota in the presence (at 24 h) or after exposure (at 48 h) to BC or S.

**Figure 2:**
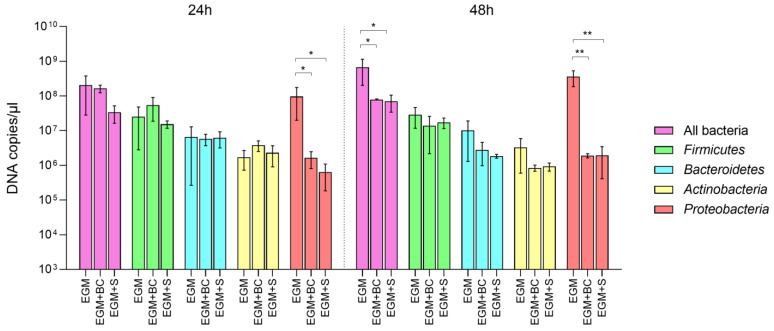
Absolute abundances (DNA copies/µL) of the total bacterial load, *Firmicutes*, *Bacteroidetes*, *Actinobacteria*, and *Proteobacteria* in fecal samples incubated for 24 h and 48 h on mucin-coated electrospun gelatin structures (EGM) in the presence of *B. cereus* vegetative cells (EGM+BC) or culture supernatant (EGM+S). * *p* < 0.05; ** *p* < 0.01.

Both BC (*p* = 0.0318) and S (*p* = 0.0295) were found to reduce the total amount of microorganisms at 48 h of co-incubation (Figure 2). The amount of *Proteobacteria* was interestingly lower (approximately 2-logs) when BC (24 h: *p* = 0.0343; 48 h: *p* = 0.0018) and S (24 h: *p* = 0.0326; 48 h: *p* = 0.0018) were present. The *Firmicutes*, *Bacteroidetes*, and *Actinobacteria* levels were not influenced by adding *B. cereus* or its supernatant.

**Figure 3:**
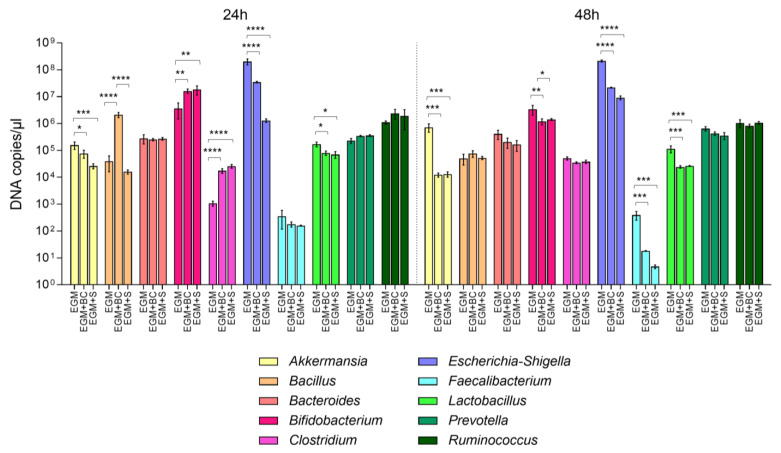
Absolute abundances (DNA copies/µL) of *Akkermansia*, *Bacillus*, *Bacteroides*, *Bifidobacterium*, *Clostridium*, *Escherichia*-*Shigella*, *Faecalibacterium*, *Lactobacillus*, *Prevotella*, and *Ruminococcus* in fecal samples incubated for 24 h and 48 h on mucin-coated electrospun gelatin structures (EGM) in the presence of *B. cereus* vegetative cells (EGM+BC) or culture supernatant (EGM+S). * *p* < 0.05; ** *p* < 0.01; *** *p* < 0.001; **** *p* < 0.0001.

More pronounced effects from adding BC or S to the gut microbiota were evidenced in selected bacterial genera (Figure 3). At 24 h, BC determined an increase in *Bacillus* in comparison with EGM controls and EGM in the presence of S (*p* < 0.0001), while S did not alter the genus levels. At the same time point, both BC and S induced higher amounts of *Bifidobacterium* (BC: *p* = 0.0090; S: *p* = 0.0033) and *Clostridium* (*p* < 0.0001) in comparison with EGM controls, while a reduction was observed for *Akkermansia* (BC: *p* = 0.0141; S: *p* = 0.0006), *Escherichia-Shigella* (*p* < 0.0001), and *Lactobacillus* (BC: *p* = 0.0126; S: *p* = 0.0379). At 48 h, the levels of *Akkermansia* (BC: *p* = 0.0002; S: 0.0002), *Bifidobacterium* (BC: *p* = 0.0089; S: *p* = 0.0159), *Escherichia-Shigella* (BC and S: *p* < 0.0001), *Faecalibacterium* (BC: *p* = 0.0003; S: 0.0002), and *Lactobacillus* (BC: *p* = 0.0002; S: 0.0003) showed a significant comparable reduction with both BC and S. *Bacteroides*, *Prevotella*, and *Ruminococcus* did not change at 24 h or 48 h when *B. cereus* was present.

### 3.3. Qualitative Analysis of Bacterial Populations by 16S rRNA Gene Sequencing

**Figure 4:**
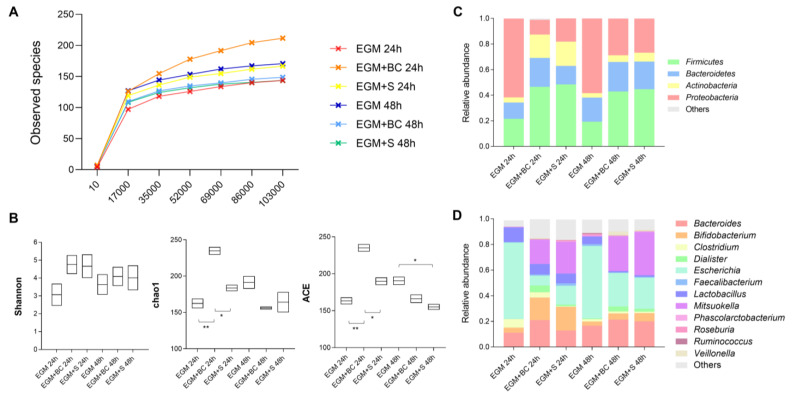
16S rRNA-based metagenomic analysis from fecal samples incubated for 24 h and 48 h on mucin-coated electrospun gelatin structures (EGM) in the presence of *B. cereus* vegetative cells (EGM+BC) or culture supernatant (EGM+S). (**A**) Rarefaction curves; (**B**) α-diversity indexes (Shannon, chao1, ACE); (**C**) Relative abundances of the main phyla; (**D**) Relative abundances of the main genera. * *p* < 0.05; ** *p* < 0.01.

16S rRNA gene metagenomic analysis of the gut microbiota grown in the presence of BC or S revealed that samples clustered closely as regards the number of identified OTUs (Figure 4A). α-diversity indexes have also been calculated (Figure 4B). Shannon index displayed no differences between EGM controls and EGM in the presence of *B. cereus* vegetative cells (EGM+BC) and supernatants (EGM+S) after 24 h and 48 h of incubation. The chao1 and ACE indexes showed EGM+BC at 24 h to be richer in OTUs than EGM (chao1: *p* = 0.0053; ACE: *p* = 0.0041) and EGM+S (chao1: *p* = 0.0144; ACE: *p* = 0.0154) at the same time point. Moreover, the ACE index of EGM at 48 h was higher than that of EGM+S (*p* = 0.0324) (Figure 4B).

**Figure 5:**
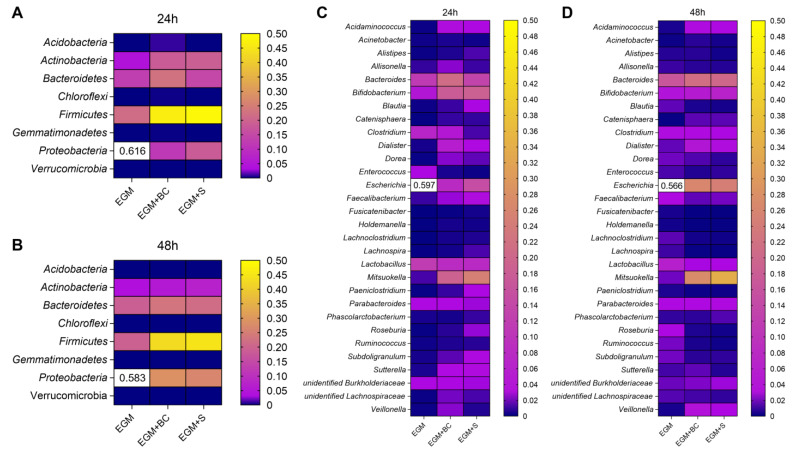
Heatmaps of the main phyla at 24 h (**A**) and 48 h (**B**) and genera at 24 h (**C)** and 48 h (**D**) from fecal samples incubated on mucin-coated electrospun gelatin structures (EGM) in the presence of *B. cereus* vegetative cells (EGM+BC) or culture supernatant (EGM+S).

Relative abundances provided a wider overview of the composition of the in vitro-grown microbial consortia. As previously observed by qPCR experiments, *Proteobacteria* drastically diminished after adding BC and S at 24 h and 48 h (Figure 4C and Figure 5A,B). The increase in *Bacteroidetes* and *Actinobacteria* evidenced in Figure 4C and Figure 5A,B was not confirmed in qPCR. *Acidobacteria*, *Chloroflexi*, *Gemmatimonadetes*, and *Verrucomicrobia* did not show alterations after adding *B. cereus* (Figure 5A,B).

**Figure 6:**
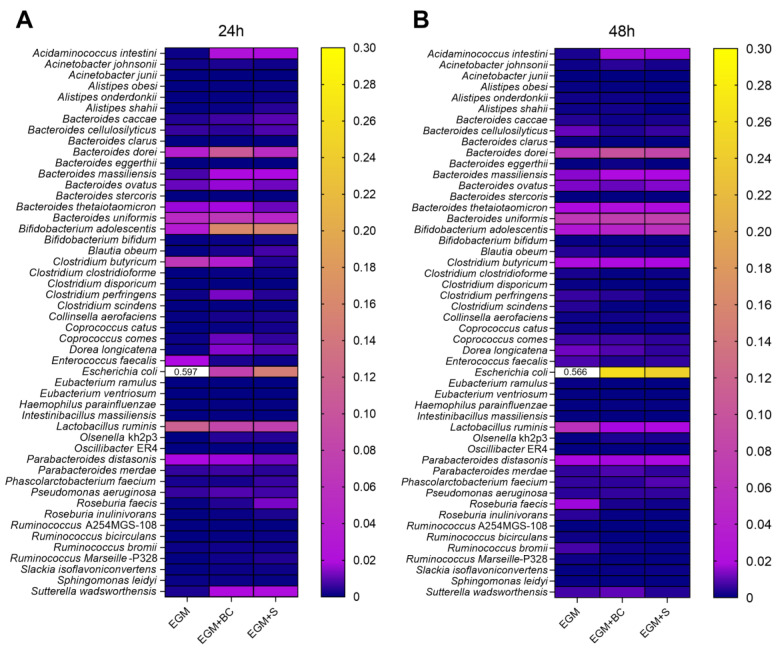
Heatmaps of the main bacterial species at 24 h (**A**) and 48 h (**B**) from fecal samples incubated on mucin-coated electrospun gelatin structures (EGM) in the presence of *B. cereus* vegetative cells (EGM+BC) or culture supernatant (EGM+S).

As regards the main genera, the abundance of *Escherichia* was significantly reduced (Figure 4D and Figure 5C,D), in accordance with the trend previously evidenced for *Proteobacteria*, to which the genus belongs. Of note, the addition of *B. cereus* vegetative cells and supernatant positively impacted the genus *Mitsuokella*, whose high expansions were observed at both 24 h and 48 h, and *Bifidobacterium*, especially at 24 h (Figure 4D and Figure 5C,D). In contrast, *Lactobacillus* levels resulted in lower levels at 24 h and 48 h in the presence of both BC and S. Heatmaps plotting the 50 more abundant species are also provided (Figure 6), pointing out *Escherichia coli* as the main species leading to a reduction in the genus *Escherichia*.

## 4. Discussion

*Bacillus cereus* is mainly recognized as the causative agent of gastrointestinal syndromes in humans due to ingesting contaminated food [11]. For this reason, we chose *B. cereus* as an infectious agent for the experiments in our 3D in vitro gut microbiota model.

*B. cereus* is known to stably interact with mucins, and several strains have already been tested for this purpose with variable results [24,25,26]. However, to the best of our knowledge, *B. cereus* ATCC 14579 has never been tested before. Our observation that this strain is able to adhere strongly to mucins is in line with the typical property of the species. Previous studies also demonstrated that important transcriptional changes occur when *B. cereus* comes in contact with mucins and that the secretion of several virulence factors is massively altered depending on mucin concentration [19,27]. Thus, it is reasonable to state that, once reaching the mucosa of the gastrointestinal tract, *B. cereus* adheres to mucins and changes its genetic expression to colonize the new environment and determine the infectious process.

In our in vitro model, adding *B. cereus* vegetative cells and supernatant caused notable alterations in the composition of the intestinal microbial consortia. No significant differences between adding vegetative cells and culture supernatants were observed. This suggests that the induced modifications of the gut microbiota were mostly due to microbial compounds secreted by the microbe in the extracellular environment. Since the effect of enterotoxigenic *B. cereus* strains on the human gut microbiota is unknown, the results obtained in this study represent a novelty in the comprehension of the intestinal *B. cereus* infection.

The finding that *B. cereus* caused the expansion of such beneficial microbes as *Bifidobacterium* was surprising. A similar result was obtained in previous studies for *Bacillus subtilis* C-3102, which was shown to secrete bifidogenic factors, allowing cross-feeding interactions and subsequent expansion of *Bifidobacterium* spp. [28,29]. The secretion of similar compounds by *B. cereus* was never investigated. However, given the prominent increase in bifidobacteria observed when microbiota was co-cultured with *B. cereus*, it is reasonable to hypothesize that *B. cereus*, similar to *B. subtilis*, could display a similar bifidogenic effect. Still, future studies are needed to address this point.

The role in human health of members belonging to the genus *Mitsuokella* (e.g., *M. jalaludinii*, *M. multacida*, and *M. dentalis*), enhanced in co-cultures with *B. cereus*, is controversial. Previous studies demonstrated that the abundance of *Mitsuokella* was reduced in several pathological conditions, such as schizophrenia (particularly in patients with low levels of serum amino acids) [30], preclinical Alzheimer’s disease [31], atrial fibrillation [32], and colon cancer [33]. Conversely, levels of *Mitsuokella* were found to be higher in obese subjects than normal-weight ones [34]. Culture supernatants of *M. jalaludinii* were also revealed to inhibit the growth and invasiveness of *Salmonella enterica* serovar Typhimurium in swine models [35]. Thus, *Mitsuokella* spp. may possess a role in intestinal health maintenance by protecting against pathogens and preventing oncogenesis. The finding that *B. cereus* could determine a significant increase in *Mitsuokella* in vitro may be interpreted as a response of the gut microbiota to counteract pathogen insertion in the microbial community, preventing the onset of infection. Further studies have to be carried out to comprehend the interactions between *B. cereus* and *Mitsuokella* spp.

*B. cereus* also determined a drastic reduction in *Proteobacteria*, *Lactobacillus,* and *Akkermansia*. Microorganisms belonging to the genera *Lactobacillus* and *Akkermansia* are important members of mucus-adherent gut communities in healthy conditions [10], and deficiencies in their amount, as in the case of the presence of *B. cereus*, are often associated with a worsening of intestinal overall wellness [36]. Conversely, the significant contextual reduction in *Proteobacteria*, mainly attributable to *E. coli*, can be interpreted as a positive effect on intestinal health since high levels of *Proteobacteria* are commonly considered microbial markers of intestinal impairment, inflammation, and diseases [37,38]. However, how *B. cereus* interacts with the abovementioned microorganisms is unknown. Antimicrobial compounds secreted by *B. cereus* ATCC 14579 (i.e., thiocillin) [39], together with tissue-degrading enzymes and cytolysins, are able to actively modify intestinal environmental niches. These factors play an important role in the colonization of the intestinal mucosa and the decimation of the microbial communities inhabiting the gut. Consequently, this leads to a very good opportunity for the pathogen to establish infection and multiply in the host.

It is also important to provide evidence that the reported alterations in the microbial composition of the gut microbiota due to infection by *B. cereus* were not restored after 24 h after the removal of the perturbing agent (i.e., vegetative cells or culture supernatant). After removing vegetative cells or the supernatant, intestinal communities do not fully recover, and eubiosis may be reached after a period of convalescence, whose duration can be variable depending on several factors.

## 5. Conclusions

In this study, *B. cereus* ATCC 14579 was shown to bind and adhere to mucins. Contacting the intestinal mucosa is probably fundamental for the microorganism to colonize the gut and display its role as an intestinal pathogen. Besides being able to cause damage to different host cells and tissues, *B. cereus* led to substantial modifications in the composition of the human gut microbiota, especially concerning *E. coli*, *Bifidobacterium*, *Lactobacillus*, *Akkermansia*, and *Mitsuokella*. In addition, perturbations of the microbiota composition were not promptly restored at the end of the infection, at least in our in vitro conditions. All these findings suggest that alterations of the intestinal microflora could be part of the pathogenic mechanism underlying *B. cereus* infection. These findings also add pieces to the complete comprehension of the pathogenic role of *B. cereus* in food poisoning and gastroenteritis.

In conclusion, the 3D in vitro model of the gut microbiota used in this study appears suitable for testing the effects of intestinal pathogens on microbial communities residing in the gut. Furthermore, it serves as an effective tool for studying the mechanisms of host damage perpetrated by pathogenic microorganisms that cause alterations in the resident microbiota.

## Data Availability

Datasets generated during the current study will be made available from the corresponding author upon reasonable request.

## Supplementary Materials

The following supporting information can be downloaded at: https://www.mdpi.com/article/10.3390/microorganisms11071826/s1, Table S1: Primer pairs used for the quantification of total bacterial load and microbial phyla; Table S2: Primer pairs used for the quantification of microbial genera.
