# Supplementary material for: Impact of Bacillus cereus on the Human Gut Microbiota in a 3D In Vitro Model
Source: Microorganisms. 2023 Jul 17;11(7):1826. doi: 10.3390/microorganisms11071826 (PMC10385275; doi:10.3390/microorganisms11071826)
Supplement: Supplementary file 1 [file microorganisms-11-01826-s001.zip › microorganisms-2508054-supplementary.pdf]

Table S1. Primer pairs used for the quantification of total bacterial load and microbial phyla [10].

Investigated bacterial group	Primer name and sequence (5′-3′)	Amplicon length (bp)	Annealing temperature (°C)
All bacteria	F: ACTCCTACGGGAGGCAGCAG<br>R: ATTACCGCGGCTGCTGG	200	60
Firmicutes	F: ATGTGGTTTAATTCGAAGCA<br>R: AGCTGACGACAACCATGCAC	126	62
Bacteroidetes	F: CATGTGGTTTAATTCGATGAT<br>R: AGCTGACGACAACCATGCAG	126	62
Actinobacteria	F: CGCGGCCTATCAGCTTGTTG<br>R: CCGTACTCCCCAGGCGGGG	600	67
Proteobacteria	F: CATGACGTTACCCGCAGAAGAAG<br>R: CTCTACGAGACTCAAGCTTGC	195	63

Table S2. Primer pairs used for the quantification of microbial genera [10].

Investigated bacterial group	Primer name and sequence (5′-3′)	Amplicon length (bp)	Annealing temperature (°C)
Akkermansia	F: CAGCACGTGAAGGTGGGGAC<br>R: CCTTGCGGTTGGCTTCAGAT	329	50
Bacillus	F: GCAACGAGCGCAACCCTTGA<br>R: TCATCCCCACCTTCCTCCGGT	92	68
Bacteroides	F: GAGAGGAAGGTCCCCCAC<br>R: CGCTACTTGGCTGGTTCAG	106	60
Bifidobacterium	F: CTCCTGGAAACGGGTGG<br>R: GGTGTTCTTCCCGATATCTACA	550	55
Clostridium	F: AAAGGAAGATTAATACCGCATAA<br>R: ATCTTGCGACCGTACTCCCC	722	57
Escherichia-Shigella	F: GAGTAAAGTTAATACCTTTGCTC<br>R: ACTCAAGCTTGCCAGTATCAG	203	52
Faecalibacterium	F: GGAGGAAGAAGGTCTTCGG<br>R: AATTCCGCCTACCTCTGCACT	248	50
Lactobacillus	F: GAGGCAGCAGTAGGGAATCTTC<br>R: GCCAGTTACTACCTCTATCCTTCTTC	126	65
Prevotella	F: GGTTCTGAGAGGAAGGTCCCC<br>R: TCCTGCACGCTACTTGGCTG	121	60
Ruminococcus	F: GGCGGCYTRCTGGGCTTT<br>R: ACCTTCCTCCGTTTTGTCAAC	451	63
